# Assessment of subventricular zone irradiation in glioblastoma patients

**DOI:** 10.3389/fonc.2026.1818058

**Published:** 2026-05-22

**Authors:** Laya K. Pillai, Maitrik J. Mehta, Ankita Parikh, Niranjan Dash, Vinay Shivhare, Sonal Patel Shah, Akash Pandya, Jayesh Kumar Singh, Krishna Prajapati, Anjali Bahuguna, Raxit Darji

**Affiliations:** Department of Radiation Oncology, The Gujarat Cancer & Research Institute, Ahmedabad, India

**Keywords:** glioblasoma multiforme, glioblastoma, neural stem/progenitor cells, radiotherapy, subventricular zone

## Abstract

**Background:**

Glioblastoma (GBM) is the most aggressive primary brain tumor in adults, with near-universal recurrence despite multimodality treatment. Increasing evidence suggests that the subventricular zone (SVZ), a major neural stem cell niche, may contribute to tumor aggressiveness, recurrence, and therapeutic resistance. However, the clinical significance of incidental SVZ irradiation remains controversial.

**Methods:**

This prospective analytical study included 28 consecutive patients with histologically confirmed GBM treated uniformly with maximal safe resection followed by temozolomide-based chemoradiation using IMRT/VMAT. Ipsilateral, contralateral, and bilateral SVZs were retrospectively contoured, and dosimetric parameters were extracted. Survival outcomes were correlated with SVZ contact and radiation dose. The primary endpoints were overall survival (OS) and progression-free survival (PFS).

**Results:**

With a median follow-up of 14.5 months, median OS and PFS were 13 months and 6.5 months, respectively. Tumors contacting the SVZ were associated with inferior OS (7 vs 15 months; p = 0.044) and shorter PFS. On univariate analysis, SVZ contact was associated with worse survival (HR 4.04). Among patients with SVZ-contacting tumors, a higher ipsilateral SVZ dose (≥52.14 Gy) showed a trend toward improved OS (p = 0.04). However, given the limited number of events, these findings should be interpreted with caution.

**Conclusion:**

SVZ contact appears to identify a higher-risk subgroup of GBM patients. Higher incidental irradiation of the ipsilateral SVZ may be associated with improved survival; however, due to the small sample size and limited events, these findings are exploratory and hypothesis-generating. Larger prospective studies are required before clinical implementation.

## Introduction

Glioblastoma (GBM) represents the most common and lethal primary malignant tumor of the central nervous system in adults, accounting for nearly 50% of all malignant gliomas (1). Despite advances in neuroimaging, molecular characterization, and multimodality treatment, the prognosis of GBM remains dismal. The current standard of care—maximal safe surgical resection followed by adjuvant radiotherapy with concurrent and maintenance temozolomide—achieves a median overall survival (OS) of only 15–18 months, with a 5-year survival rate of less than 10% (2, 3). Tumor recurrence is nearly universal and is predominantly local, occurring within the high-dose radiation field, underscoring the intrinsic radioresistance and aggressive infiltrative nature of this disease (4).

A growing body of evidence suggests that tumor recurrence and treatment resistance in GBM are driven by a subpopulation of cells known as glioma stem cells (GSCs). These cells possess stem-like properties, including self-renewal, multipotency, and resistance to radiation and chemotherapy, and are believed to play a critical role in tumor initiation, maintenance, and recurrence (5, 6). Importantly, GSCs share phenotypic and molecular similarities with normal neural stem cells (NSCs), raising the possibility that GBM may originate from or be sustained by neural stem cell niches within the adult brain (7).

The subventricular zone (SVZ), lining the lateral ventricles, is the largest and most well-characterized neural stem cell niche in the adult human brain. It harbors astrocyte-like NSCs capable of proliferation, migration, and differentiation throughout life (8). Preclinical studies have demonstrated that NSCs within the SVZ are susceptible to oncogenic transformation and can give rise to glioma-like tumors when exposed to genetic insults commonly observed in GBM, such as TP53 loss and EGFR amplification (9, 10). Furthermore, experimental models suggest that GSCs residing within or migrating to the SVZ may be shielded from therapeutic insult, thereby serving as a reservoir for tumor repopulation following standard treatment (11).

Clinical observations support the biological relevance of the SVZ in GBM behavior. Several imaging-based studies have shown that GBMs contacting the SVZ exhibit more diffuse infiltration patterns, increased contralateral spread, and a higher likelihood of multifocal disease at diagnosis (12, 13). SVZ involvement has also been associated with inferior progression-free survival (PFS) and OS, independent of other known prognostic factors such as age, extent of resection, and performance status (14). These findings suggest that the SVZ may function not merely as an anatomical structure but as an active participant in GBM pathogenesis and progression.

Given this biological and clinical context, interest has emerged in exploring whether irradiation of the SVZ during postoperative radiotherapy could improve oncological outcomes by targeting resident NSCs and GSCs. Retrospective dosimetric analyses have suggested that higher radiation doses delivered incidentally to the ipsilateral SVZ are associated with improved PFS and, in some studies, OS (15, 16). Evers et al. demonstrated that patients receiving ≥40 Gy to the ipsilateral SVZ had significantly longer PFS compared to those receiving lower doses (17). Similar observations have been reported in other institutional series, reinforcing the hypothesis that SVZ irradiation may suppress stem-cell–mediated tumor regrowth (18).

However, the clinical benefit of SVZ irradiation remains controversial. Several large retrospective studies have failed to demonstrate a consistent survival advantage, and concerns have been raised regarding potential neurocognitive toxicity due to irradiation of a critical stem cell niche involved in neurogenesis (19, 20). Moreover, heterogeneity in SVZ delineation, radiation dose thresholds, laterality (ipsilateral vs bilateral SVZ), and study design has limited the generalizability of existing evidence (21). Prospective trials specifically designed to assess the impact of intentional SVZ targeting are scarce, and current clinical guidelines do not mandate routine inclusion of the SVZ in GBM radiotherapy target volumes (22).

A better understanding of the relationship between SVZ dose and clinical outcomes may help refine radiotherapy planning strategies and contribute to personalized treatment approaches in GBM. The present study aims to evaluate the correlation between incidental subventricular zone (SVZ) irradiation dose and its impact on recurrence and survival in a homogeneous cohort of glioblastoma patients, while exploring the feasibility and potential therapeutic benefit of deliberate SVZ inclusion in radiotherapy planning to reduce recurrence and improve survival outcomes.

## Materials and methods

### Study design and patient population

This was a single-arm, prospective analytical study conducted in the Department of Radiation Oncology at The Gujarat Cancer and Research Institute (GCRI), BJ Medical College, Ahmedabad. Patients were enrolled between June 2022 and July 2024. This study was conducted in accordance with institutional ethical standards. All patient data were anonymized prior to analysis. Written informed consent was obtained from all participants prior to enrollment.

A total of 29 consecutive patients with histopathologically confirmed glioblastoma (WHO grade IV) who presented to the Radiation Oncology outpatient department for postoperative radiotherapy were included. Eligible patients were aged between 18 and 70 years and had a Karnofsky Performance Status (KPS) score ≥70.

### Inclusion and exclusion criteria

Inclusion criteria were: (1) age 18–70 years; (2) KPS ≥70; (3) postoperative status following gross total resection (GTR) or subtotal resection (STR); (4) tumor location in the frontal, temporal, or parietal lobes; and (5) planned treatment with conformal radiotherapy techniques including intensity-modulated radiotherapy (IMRT) or volumetric-modulated arc therapy (VMAT/RapidArc).

Exclusion criteria included: (1) low-grade glioma; (2) inoperable tumors; (3) age <18 years or >70 years; and (4) KPS <70.

### Simulation and immobilization

All patients were immobilized in the supine position with arms placed alongside the body using a three-clamp thermoplastic mask (ORFIT) secured to a Perspex baseplate and appropriate headrest. The head was positioned in slight extension with shoulders pulled inferiorly to optimize reproducibility. This immobilization setup was used consistently for both computed tomography (CT) and magnetic resonance imaging (MRI) simulations.

### MRI simulation

MRI simulation was performed using a 3-Tesla scanner (Siemens Magnetom Skyra, Germany) with a standard flex head coil. A custom Perspex baseplate and headrest were used to secure the thermoplastic mask during MRI acquisition. Turbo spin-echo sequences were used to minimize image distortion. Imaging was obtained from the vertex to the skull base, including T1-weighted pre- and post-gadolinium contrast, T2-weighted, and FLAIR sequences with a slice thickness of 3 mm. Additionally, T1 MP-RAGE sequences were acquired at 1-mm thickness and reconstructed to 3-mm slices. MRI images were transferred to the Monaco treatment planning system (TPS) via DICOM.

### CT simulation

CT simulation was performed on a flat tabletop CT simulator (Siemens, Germany) with patients positioned identically to the MRI setup. Laser alignment points were marked using lead fiducial markers at the glabella and bilateral temporal regions to facilitate accurate image registration and treatment setup. Following intravenous administration of iodinated contrast (Iohexol), axial CT images with 3-mm slice thickness were acquired from the vertex to the skull base. Images were reviewed for positioning accuracy and transferred to the Monaco TPS.

### CT–MRI image fusion

CT and MRI datasets were imported into the TPS and fused using automated rigid registration. Registration accuracy was verified by visual assessment of anatomical landmarks, particularly the lateral ventricles and brainstem. Both T1- and T2-weighted MRI sequences were utilized to optimize target delineation.

### Target volume delineation

Target volumes were contoured on fused CT–MRI images in accordance with ICRU Reports 50 and 62 and the NRG glioblastoma protocol. Gross tumor volume (GTV) was defined based on postoperative resection cavity and residual enhancing disease on contrast-enhanced T1-weighted MRI (GTV2). A second GTV (GTV1) encompassing the hyperintense region on T2/FLAIR imaging was delineated to include peritumoral edema.

GTV1 was expanded isotropically by 2 cm to create clinical target volume 1 (CTV1), limited by anatomical barriers such as the falx, ventricles, and skull. CTV1 was copied and trimmed to generate the phase I treatment volume (CTV_46). Similarly, GTV2 was expanded by 2 cm to create CTV2, which was subsequently trimmed to form the phase II treatment volume (CTV_14). Planning target volumes (PTVs) were generated by adding a uniform 5-mm margin to each respective CTV (PTV_46 and PTV_14).

All contouring was performed by a single investigator to minimize interobserver variability and was independently reviewed and approved by an experienced radiation oncologist and, when required, by a neuroradiologist, each with over 10 years of clinical experience.

### Subventricular zone delineation

The ipsilateral, contralateral, and bilateral subventricular zones (SVZs) were contoured retrospectively on the planning CT images using co-registered MRI for anatomical reference. The SVZ was defined as a 5-mm periventricular region along the lateral wall of the lateral ventricles, including the temporal horns, consistent with established definitions in prior literature. The ipsilateral SVZ (iSVZ) was defined as the SVZ on the same side as the tumor; in multifocal or midline tumors, laterality was assigned based on the dominant tumor bulk. SVZ contours were reviewed and approved by an experienced radiation oncologist and neuroradiologist.

Dose–volume histograms (DVHs) were generated automatically, and mean and median doses to the ipsilateral, contralateral, and bilateral SVZs were recorded.

### Radiotherapy planning and delivery

All patients were treated with conformal radiotherapy using IMRT or VMAT/RapidArc techniques. Treatment planning was performed using Monaco and/or Eclipse TPS. The prescribed dose was 60 Gy delivered in 30 fractions over 6 weeks: 46 Gy in 23 fractions to PTV_46 (phase I), followed by a boost of 14 Gy in 7 fractions to PTV_14 (phase II), with one fraction per day, five days per week.

Plan evaluation adhered to institutional dose-volume constraints: PTV V95% ≥95%, CTV V98% ≥98%, and GTV V100% = 100%. All plans underwent standard quality assurance procedures prior to treatment delivery.

### Organs at risk

Dose constraints for organs at risk were as follows: brainstem, optic nerves, and optic chiasm (Dmax <54 Gy); spinal cord and eyeballs (Dmax <45 Gy); lenses (Dmean <6 Gy); and cochleae/middle ears (Dmean <23 Gy).

### Concurrent chemotherapy and image guidance

All patients received concurrent temozolomide chemotherapy (75 mg/m²/day), initiated on the first day of radiotherapy. Patients were instructed to take temozolomide 30–60 minutes prior to each radiotherapy fraction. Weekly cone-beam CT (CBCT) imaging was performed for image guidance. Two patients required repeat simulation and replanning due to anatomical changes observed during treatment.

### Follow-up and outcome assessment

Patients were followed clinically and radiologically with contrast-enhanced MRI at 1 month post-radiotherapy and at 3-monthly intervals thereafter until disease progression or death. Median follow-up duration was 14.5 months, calculated from the date of surgery to last follow-up or death.

Disease recurrence was defined as radiographic progression on contrast-enhanced MRI or CT, as confirmed by a multidisciplinary tumor board. Pseudoprogression was managed with serial imaging to confirm true progression.

The primary endpoints were progression-free survival (PFS) and overall survival (OS). PFS was defined as the interval from the date of primary surgery (or initiation of treatment) to documented disease progression or death. OS was defined as the time from pathological diagnosis to death from any cause. Patients without events were censored at last follow-up.

### Statistical analysis

SVZ incidental dose parameters, including mean (D mean) and median (D median) doses to the ipsilateral, contralateral, and bilateral SVZs, were recorded. Data were entered into Microsoft Excel and analyzed using SPSS version 26 (IBM Corp., Armonk, NY, USA).

Overall survival (OS) and progression-free survival (PFS) were estimated using the Kaplan–Meier method, and comparisons between groups were performed using the log-rank test. A p-value ≤ 0.05 was considered statistically significant. Univariate analysis was conducted to evaluate the association between clinical, dosimetric, and treatment-related variables and survival outcomes.

The dose cut-offs were derived from cohort-based central tendency measures due to the limited sample size. Specifically, the ipsilateral SVZ threshold (52.14 Gy) represents the mean value of the median dose (Dmedian) across the cohort, while the bilateral SVZ threshold (45.11 Gy) corresponds to the median of the cohort distribution. Although statistically optimized approaches such as receiver operating characteristic (ROC) curve analysis or minimum p-value methods are often preferred, these were not feasible due to the small number of events.

Therefore, the selected thresholds should be considered pragmatic and exploratory, intended to allow preliminary assessment of dose–response relationships rather than definitive cut-off determination.

## Results

### Patient characteristics

A total of 28 patients met all inclusion and exclusion criteria and were included in the final analysis. The median age at diagnosis was 45 years (range: 35–59 years). All patients had a Karnofsky Performance Status (KPS) ≥70 and underwent surgical resection, either gross total resection (GTR) or subtotal resection (STR). All but one patient completed the planned course of postoperative radiotherapy with concurrent temozolomide; this patient experienced clinical deterioration during treatment. MGMT promoter methylation status was not available for all patients; however, all except one received adjuvant temozolomide chemotherapy as per standard protocol. The median follow-up duration was 14.5 months. Baseline demographic and clinical characteristics are summarized in **Tables 1**, **2**.

**Table 1:** Baseline demographic and clinical characteristics of the study cohort (N = 28).

Characteristic	Value
Median age, years (range)	45 (35–59)
KPS ≥70	28 (100%)
Extent of surgery (GTR/STR)	28 (100%)
SVZ contact at diagnosis	15 (53.6%)
No SVZ contact	13 (46.4%)
Completion of concurrent chemoradiation	27 (96.4%)
Adjuvant temozolomide received	27 (96.4%)
Median follow-up, months	14.5

**Table 2:** Baseline demographic, clinical, molecular, and treatment characteristics of the study cohort of glioblastoma patients.

Variable	Number (%)
AGE (years)	
< 45	15 (53.57%)
> 45	13 (46.4%)
GENDER	
Female	10 (35.7%)
Male	18 (64.28%)
IDH STATUS	
Wild type	20 (71.4%)
Mutant	4 (14.28%)
Not done	4 (14.28%)
SVZ CONTACTING	
Yes	15 (53.57%)
No	13 (46.4%)
GROUPING	
I	11 (39.28%)
II	4 (14.28%)
III	13 (46.4%)
IV	0 (0%)
LOCATION	
Frontal	9 (32.14%)
Occipital	3 (10.7%)
Parietal	8 (28.57%)
Suprasellar	1 (0.03%)
Temporal	6 (21.4%)
Thalamic	1 (0.03%)
ADJUVANT CHEMOTHERAPY	
Yes	27 (96.42%)
No	1 (0.03%)

### Survival outcomes

At the time of analysis, 24 patients were alive and 4 deaths had occurred, corresponding to an overall survival rate of 85.7%. Disease progression was observed in 10 patients (35.7%), while 18 patients (64.3%) remained progression-free. The median overall survival (OS) for the entire cohort was 13 months (range: 4.6–18.35 months), and the median progression-free survival (PFS) was 6.5 months (range: 1.44–14 months). This estimate has been interpreted cautiously due to the limited number of death events. Survival outcomes for the overall cohort are detailed in **Table 3**.

**Table 3:** Survival outcomes of the entire cohort.

Outcome	Value
Median OS, months (range)	13 (4.6–18.35)
Median PFS, months (range)	6.5 (1.44–14)
Alive at last follow-up	24 (85.7%)
Deaths	4 (14.3%)
Overall survival rate	85.7%
Progression observed	10 (35.7%)
Progression-free rate	64.3%

### Impact of SVZ contact on survival

**Figure 1:**
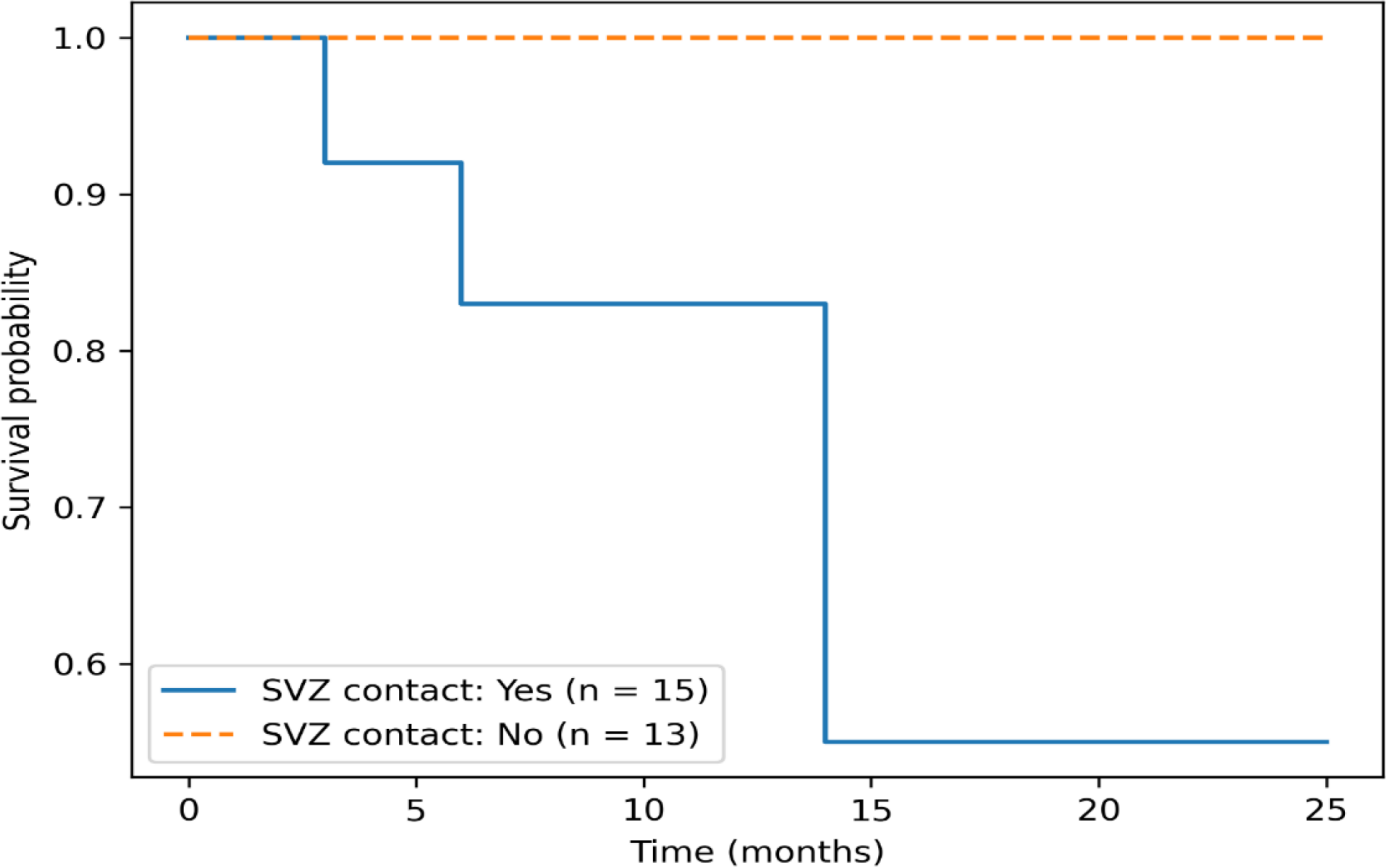
Kaplan–Meier overall survival curves stratified by SVZ contact status. Patients with SVZ-contacting tumors demonstrated inferior survival compared to those without SVZ contact. Median overall survival was 7 months in the SVZ-contact group, while median survival in the non-contact group was not reached due to absence of events during follow-up.

Tumors in contact with the subventricular zone (SVZ) at diagnosis were associated with inferior survival outcomes. Patients with SVZ-contacting tumors had a median OS of 7 months. Median overall survival in the non-SVZ-contact group could not be reliably estimated due to absence of events during the follow-up period. Therefore, survival comparisons should be interpreted cautiously (p = 0.044). Median PFS was also shorter in the SVZ-contacting group (5 months vs 8 months). These findings are summarized in **Table 4** and **Figure 1**.

**Table 4:** Survival outcomes according to SVZ contact.

SVZ contact status	N	Deaths	Median OS (months)	Median PFS (months)
Yes	15	4	7	5
No	13	0	15	8
	Total N	Deaths	Alive	Survival rate
YES	15	4	11	73.30%
NO	13	0	13	100.00%

Median OS in the non-SVZ-contact group is not estimable due to absence of death events during follow-up.

### Univariate analysis of prognostic factors

On univariate analysis, SVZ contact at presentation was associated with worse overall survival (p = 0.044; hazard ratio [HR] 4.04), although this finding should be interpreted cautiously given the limited number of events. Receipt of adjuvant chemotherapy was associated with a statistically significant OS benefit (p = 0.025). Additionally, superior target volume coverage showed a trend toward improved OS: patients with PTV V100 >99.625% and CTV V98 >98.235% demonstrated significantly better survival outcomes (both p = 0.03). Tumor location influenced PFS, with frontal, parietal, and occipital tumors showing higher PFS rates compared with temporal, thalamic, and suprasellar tumors; however, this difference did not reach statistical significance. Univariate analysis results are presented in **Table 5**.

**Table 5:** Univariate analysis of factors affecting overall survival.

Variable	Comparison	p-value	Hazard ratio
SVZ contact	Yes vs No	0.044	4.04
Adjuvant chemotherapy	Yes vs No	0.025	—
PTV coverage (V100)	>99.625% vs ≤99.625%	0.03	—
CTV coverage (V98)	>98.235% vs ≤98.235%	0.03	—
Tumor location	Superficial vs deep	NS	—

### SVZ dosimetric analysis and survival correlation

Mean and median doses to the ipsilateral, contralateral, and bilateral SVZs were calculated. The mean D_median to the ipsilateral SVZ was 52.14 Gy, while the mean D_mean was 50.7 Gy. The median D_median and D_mean were 51.43 Gy and 49.27 Gy, respectively. Dosimetric parameters are summarized in **Table 6**.

**Table 6:** SVZ dosimetric parameters.

SVZ region	Dosimetric parameter	Mean dose (Gy)	Median dose (Gy)
Ipsilateral SVZ	D_mean	50.7	49.27
Ipsilateral SVZ	D_median	52.14	51.43
Bilateral SVZ	D_median (threshold)	—	45.11

In patients with SVZ-contacting tumors, an ipsilateral SVZ D_median ≥52.14 Gy was associated with a trend toward improved OS (p = 0.04), although this observation is limited by small sample size and number of events. Similarly, a bilateral SVZ D_median ≥45.11 Gy was associated with a possible OS benefit (p = 0.04), which should be interpreted cautiously given the limited statistical power. These associations are detailed in **Table 7**.

**Table 7:** Impact of SVZ irradiation dose on overall survival.

SVZ dose parameter	Cut-off value	Survival impact	p-value
Ipsilateral SVZ D_median	≥52.14 Gy	Improved OS	0.04
Bilateral SVZ D_median	≥45.11 Gy	Improved OS	0.04

### Progression-free survival by SVZ contact

**Figure 2:**
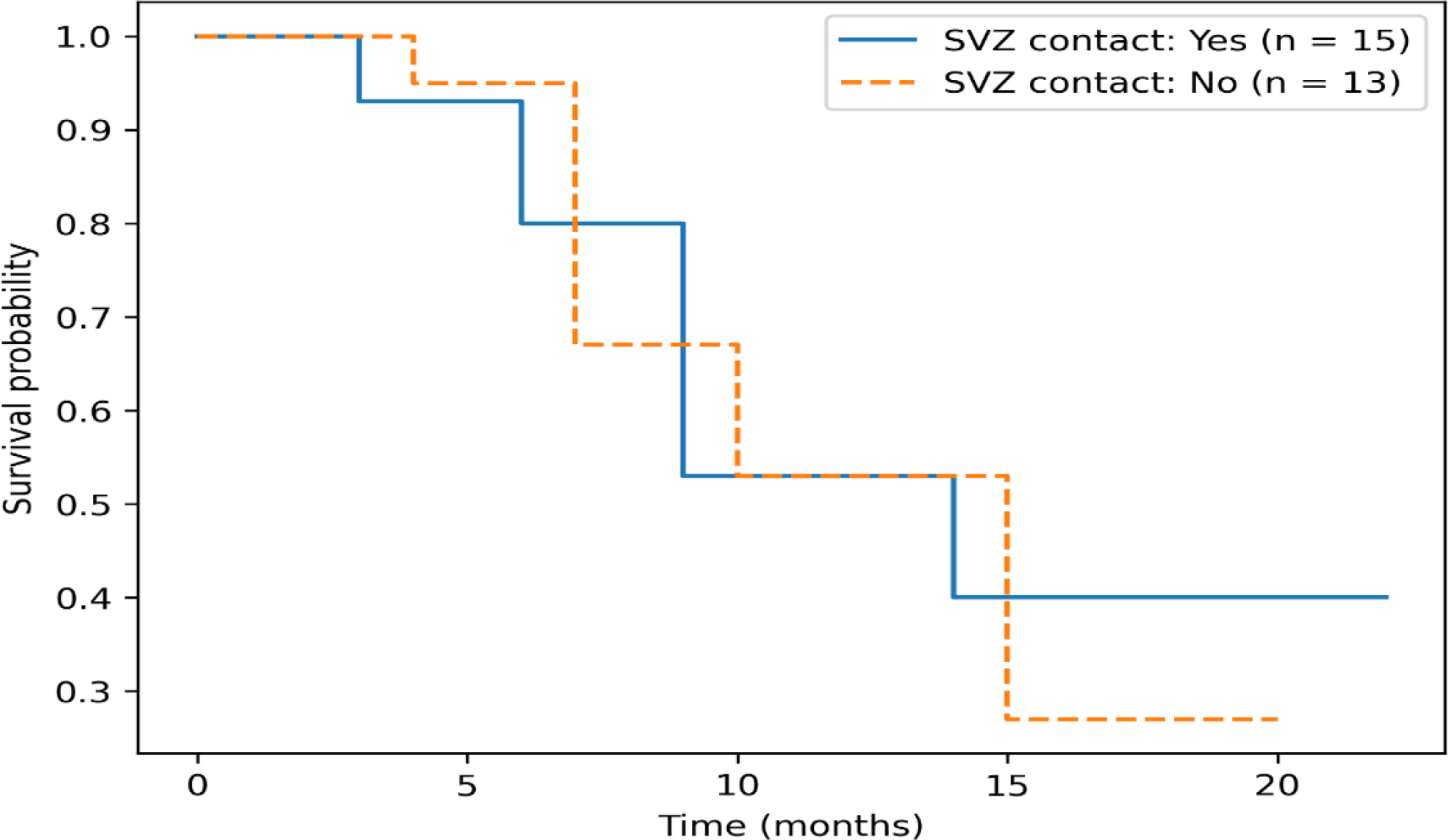
Kaplan–Meier progression-free survival curves according to SVZ contact status. No statistically significant difference in progression-free survival was observed between patients with and without SVZ contact.

Progression-free survival rates were comparable between SVZ-contacting and non-contacting tumors, with PFS rates of 66.7% and 61.5%, respectively. These findings are summarized in **Table 8** and **Figure 2**.

**Table 8:** Progression-free survival according to SVZ contact.

SVZ contact status	N	Progression	No progression	PFS rate (%)
Yes	15	5	10	66.7
No	13	5	8	61.5

### Statistical limitations

Due to the limited number of events (n = 4 deaths) and small subgroup sizes, particularly within stratified analyses, the statistical reliability of subgroup comparisons is inherently constrained. Therefore, associations observed in SVZ dose-response analyses and subgroup survival comparisons should be interpreted as exploratory and hypothesis-generating rather than definitive.

## Discussion

The optimal management of neurogenic regions, particularly the subventricular zone (SVZ), in glioblastoma remains controversial due to the complex biological interplay between neural stem cells and brain tumor stem cells. In this prospective, homogeneous cohort of glioblastoma patients treated with uniform surgery and standard temozolomide-based chemoradiation, we evaluated the prognostic significance of SVZ involvement and the potential impact of incidental SVZ irradiation on survival outcomes.

A major strength of the present study lies in its clinical homogeneity. All patients underwent maximal safe resection followed by adjuvant chemoradiation with identical dose and fractionation schedules, thereby minimizing treatment-related confounding factors. Tumor delineation and treatment planning were rigorously cross-checked by experienced radiation oncologists, ensuring consistency and accuracy. Although MGMT methylation status was unavailable in a subset of patients, other established prognostic variables were uniformly assessed.

Our findings demonstrate that initial tumor contact with the SVZ is a significant adverse prognostic factor, associated with inferior overall survival (OS) and progression-free survival (PFS). This observation is consistent with pioneering work by Lim et al. (12) and subsequent studies that have identified SVZ involvement as a marker of aggressive tumor biology. Glioblastomas in contact with the SVZ are hypothesized to originate from or recruit neural stem-like cells, conferring increased migratory capacity, invasiveness, and therapeutic resistance.

From a biological standpoint, experimental data suggest that neuronal progenitor cells residing in the SVZ possess greater migratory potential than those from non-SVZ regions, providing a plausible explanation for the higher incidence of distant and multifocal recurrences observed in SVZ-contacting tumors. Our study further supports this concept, as all disease-specific deaths and early progressions occurred in patients with SVZ-contacting tumors.

In univariate analysis, higher incidental radiation dose to the ipsilateral SVZ—particularly a median dose ≥52.14 Gy—showed a trend toward improved OS in patients with SVZ-contacting tumors. Although this OS benefit did not translate into a consistent PFS advantage, likely due to the short median follow-up and small sample size, the trend aligns with several prior reports suggesting that adequate SVZ irradiation may help eradicate glioma stem-cell niches.

Importantly, previously proposed dose thresholds of 43 Gy and 59.1 Gy did not demonstrate statistically significant survival benefits in our cohort. This discrepancy may reflect differences in patient selection, extent of resection, follow-up duration, and SVZ delineation methods across studies. Nevertheless, our findings reinforce the notion that the ipsilateral SVZ dose, rather than bilateral or contralateral SVZ irradiation, is most relevant for oncologic outcomes.

Several retrospective and pooled analyses have evaluated the role of SVZ irradiation with heterogeneous results. These studies are summarized in **Table 9**. Evers et al. (17) reported improved PFS with mean bilateral SVZ doses >43 Gy, whereas Lee et al. (23) and Gupta et al. (24) identified improved PFS and OS, respectively, with higher ipsilateral SVZ doses (>58–59 Gy). Chen et al. (25) showed a trend toward restricted to patients undergoing gross total resection, highlighting the importance of minimizing residual disease when targeting putative stem-cell niches.

**Table 9:** The summary of all major studies of SVZ radiation.

Researcher	Size of experiment	Surgery	Chemotherapy	Radiotherapy	Result
Huang et al.	176 patients	+	Partly	+	SVZ contact (P = 0.008) was significantly associated with a shortened recurrence time
Evers et al.	55 patients	+	+ (except one)	External beam radiation therapy (except one)	Bilateral SVZ received greater than the median SVZ dose (43 Gy) had a significant improvement in PFS
Lee et al.	173 patients	+	–	+	High radiation therapy doses to ipsilateral SVZ remained an independent predictor of improved PFS but not of OS
Chen et al.	116 patients	+	+	Intensity-modulated radiation therapy (IMRT) (60 Gy/30 f)	iSVZ dose was greater than 40 Gy; both PFS and OS improved in patients with GBM after GTR
Luchi et al.	Single-institution prospective study	+	+	Hypofractionated high-dose IMRT	Hypofractionated radiation (PTV1 = 68 Gy/8 f) had satisfactory results in local control and survival
Darázs et al.	41 patients	+	–	+	Higher mean dose (≥58 Gy) to the iSVZ had significantly better OS
Adeberg et al.	607 patients	68.5%	71.7%	28.3%	GBM close to the SVZ has decreased survival and a higher risk of multifocal or distant progression
Gupta et al.	40 patients	+	+	Three-dimensional conformal radiation therapy (3D-CRT, 60 Gy/30 f)	Mean dose of iSVZ greater than 59.9 Gy was an independent factor of OS
Elicin et al.	60 patients	+	+	3D-CRT (60 Gy/30 f)	Higher cSVZ dose (>59.2 Gy) had a negative effect on OS and PFS
Bender et al.	200 patients	+	+	IMRT	Ipsilateral or contralateral SVZ dose had no significant effect on OS and PFS
Weinberg et al.	50 patients	+	+	External beam radiation therapy (60 Gy/30 f)	Distant SVZ site receiving ≤45 Gy had the shortest survival
Mathew et al.	47 patients	+	+	+	iSVZ dose ≥56 Gy trended toward improved OS and PFS; cSVZ dose ≥50 Gy appeared to have better OS and PFS

Conversely, some studies have suggested worse outcomes with higher contralateral SVZ doses, potentially reflecting larger tumor volumes, midline crossing, and limited surgical resection rather than a direct detrimental effect of irradiation. In our cohort, contralateral SVZ dose was not associated with survival, supporting selective ipsilateral SVZ targeting rather than routine bilateral inclusion. A comparative summary of published SVZ dosimetric studies is presented in **Table 10**.

**Table 10:** Comparison of subventricularzone(SVZ) dose thresholds, volumetric parameters, and survival outcomes (progression-free survival and overall survival) reported in the literature and observed in the current study of glioblastoma patients.

Study (author, year)	Sample size	SVZ threshold dose	iSVZ volume (cc)	cSVZ volume (cc)	bSVZ volume (cc)	iSVZ Dose (Gy)	cSVZ dose (Gy)	bSVZ dose (Gy)	PFS (median, months)	PFS *p*-value	OS (median, months)	OS *p*-value
Evers et al., 2010 (17)	55	>43 Gy	5.05	–	–	46 ± 15.5	41 ± 16.1	–	15 vs 7.2	0.03	–	–
Gupta et al., 2012 (15)	40	iSVZ >58 Gy	5.6 ± 2.5	6.4 ± 3	–	58.7	53.6	56.2	11 (8.9–13) vs 11	0.92	17 (11.6–22.4) vs 15	0.95
Lee et al., 2013 (18)	173	iSVZ >59.4 Gy	4.3 ± 1.3	5 ± 1.6	–	49.2	35.2	60.1	10.4 (0.1–71.3)	0.042	19.6 (4.4–104)	0.173
Chen et al., 2013 (16)	116	iSVZ >40 Gy	7.05 (2.99–42)	7.91 (4.18–46)	14.76 (5.37–28.3)	48.7 (19.6–60)	34.4 (15.9–60)	–	4.5 (1.7–60)	0.434	–	0.754
Elicin et al., 2014 (26)	60	iSVZ >59.2 Gy	5.4 ± 2.4	6.4 ± 2.3	11.8 ± 4.2	58.8 ± 6.5	44.9 ± 15.9	51.9 ± 10.4	9.5 (7.1–11.1) vs 7.1	0.018	19.2 (12.7–25.3)	0.004
Adeberg et al., 2014 (13)	65	iSVZ >40 Gy	14.05 (8.41–28)	14.5 (8.82–28)	–	58.7	53.6	6.2	11 (8.9–13) vs 11	0.02	17 (11.6–22.4) vs 15	0.95
Kazmi et al., 2014 (27)	72	iSVZ 70 (EQD2) vs 60	–	–	–	60.6 (33.4–69.8)	39.5 (19.4–61.2)	49.1 (29.3–64.3)	7.1 (5.8–9.6) vs 11	0.052	15.2 (11.8–16) vs 18.4	NS
Khalifa et al., 2016 (28)	43	iSVZ >40 Gy	5.13 ± 1.1	5.5 (3.4–9.6)	10.6 (6.2–20.6)	51.3 (19.6–61.4)	15.4 (4.4–48.7)	35 (10.9–51.8)	6.5 (4.4–9.3)	–	22.7 (14.5–26.2)	–
Our Study, 2024	28	iSVZ >52.14 Gy	7.9 (6.02–10.39)	7.89 (5.80–10.03)	15.57 (11.61–27.99)	50.7	38.8	44.98	6.5 (4.4–14.4) vs 6	0.78	13 (4.6–18.3)	0.04

Our observations suggest that SVZ-contacting tumors represent a biologically and clinically high-risk subgroup of glioblastoma. In such cases, deliberate inclusion of the entire ipsilateral SVZ in the clinical target volume may be justified to mitigate the risk of tumor repopulation from untreated stem-cell reservoirs. Limiting irradiation to only the involved SVZ segment may leave adjacent neurogenic niches untreated, potentially facilitating recurrence.

Conversely, in non-SVZ-contacting tumors, we observed no survival benefit from higher SVZ doses. Given the risk of radiation-induced neurotoxicity, routine SVZ irradiation in this subgroup appears unwarranted and should be avoided. Tumor location also influenced outcomes in our cohort, with temporal, thalamic, and suprasellar tumors demonstrating poorer PFS compared to frontal, parietal, and occipital tumors. This finding likely reflects proximity to the SVZ and deeper eloquent structures, further emphasizing the importance of anatomical considerations in risk stratification.

Finally, the survival benefit associated with adjuvant chemotherapy observed in our study is consistent with established landmark trials, reinforcing adherence to standard-of-care treatment protocols. Despite its prospective design and treatment uniformity, this study is limited by its small sample size, relatively short follow-up, and incomplete MGMT methylation data. These factors may have limited the detection of statistically significant differences in PFS and precluded robust multivariate modeling for all variables.

The most important limitation of this study is the small sample size and low number of survival events, which significantly limits statistical power and reliability of survival estimates. In particular, the absence of events in certain subgroups precludes accurate estimation of median survival and may introduce bias. Therefore, findings should be interpreted as hypothesis-generating rather than definitive evidence.

## Conclusion

In conclusion, SVZ-contacting glioblastomas appear to represent a higher-risk subgroup with inferior survival outcomes. Higher ipsilateral SVZ radiation dose showed a potential association with improved survival in this subgroup. However, given the small sample size, limited number of events, and absence of complete molecular data, these findings should be interpreted with caution. This study should be considered exploratory and hypothesis-generating. Larger prospective studies are required to validate these observations before recommending routine inclusion of the SVZ in radiotherapy planning.

Our results suggest a potential role of ipsilateral SVZ targeting in selected patients; however, this requires validation in larger prospective studies before clinical adoption. Nevertheless, given the exploratory nature of this analysis and its inherent limitations, deliberate SVZ irradiation cannot yet be recommended as a standard treatment strategy. Further large-scale, prospective randomized controlled trials are essential to validate the role of prophylactic ipsilateral SVZ irradiation and to define optimal dose thresholds. In this context, intensity-modulated radiation therapy (IMRT) may provide a feasible approach to safely incorporate SVZ targeting while sparing critical neurocognitive structures. Ongoing and recently completed clinical trials, including NCT01478854, NCT02039778, and NCT02177578, are expected to provide more definitive evidence regarding the therapeutic value of SVZ modulation in glioblastoma and may ultimately inform future treatment paradigms for this highly aggressive disease.

## Data Availability

The raw data supporting the conclusions of this article will be made available by the authors, without undue reservation.

## References

[1] Ostrom QT Gittleman H Liao P Vecchione-Koval T Wolinsky Y Kruchko C . CBTRUS statistical report: Primary brain and other central nervous system tumors diagnosed in the United States in 2013–2017. Neuro Oncol. (2020) 22:iv1–iv96. doi:10.1093/neuonc/noaa200. PMID: 33123732 PMC7596247

[2] Stupp R Mason WP van den Bent MJ Weller M Fisher B Taphoorn MJB . Radiotherapy plus concomitant and adjuvant temozolomide for glioblastoma. N Engl J Med. (2005) 352:987–96. doi:10.1056/nejmoa043330. PMID: 15758009

[3] Stupp R Taillibert S Kanner AA Read W Steinberg DM Lhermitte B . Effect of tumor-treating fields plus maintenance temozolomide vs temozolomide alone on survival in patients with glioblastoma. JAMA. (2017) 318:2306–16. doi:10.1001/jama.2017.18718. PMID: 29260225 PMC5820703

[4] Brandes AA Tosoni A Franceschi E Sotti G Frezza G Amista P . Recurrence pattern after temozolomide concomitant with and adjuvant to radiotherapy in newly diagnosed patients with glioblastoma. Cancer. (2009) 115:4956–63. doi:10.1002/cncr.24406. PMID: 19188675

[5] Singh SK Hawkins C Clarke ID Squire JA Bayani J Hide T . Identification of human brain tumour initiating cells. Nature. (2004) 432:396–401. doi:10.1038/nature03128. PMID: 15549107

[6] Bao S Wu Q McLendon RE Hao Y Shi Q Hjelmeland AB . Glioma stem cells promote radioresistance by preferential activation of the DNA damage response. Nature. (2006) 444:756–60. doi:10.1038/nature05236. PMID: 17051156

[7] Sanai N Alvarez-Buylla A Berger MS . Neural stem cells and the origin of gliomas. N Engl J Med. (2005) 353:811–22. doi:10.1056/nejmra043666. PMID: 16120861

[8] Quiñones-Hinojosa A Sanai N Soriano-Navarro M Gonzalez-Perez O Mirzadeh Z Gil-Perotin S . Cellular composition and cytoarchitecture of the adult human subventricular zone. J Comp Neurol. (2006) 494:415–34. doi:10.1093/neurosurgery/57.2.427a 16320258

[9] Alcantara Llaguno S Chen J Kwon CH Jackson EL Li Y Burns DK . Malignant astrocytomas originate from neural stem/progenitor cells in a somatic tumor suppressor mouse model. Cancer Cell. (2009) 15:45–56. doi:10.1016/j.ccr.2008.12.006. PMID: 19111880 PMC2650425

[10] Zhu Y Guignard F Zhao D Liu L Burns DK Mason RP . Early inactivation of p53 tumor suppressor gene cooperating with NF1 loss induces Malignant astrocytoma. Cancer Cell. (2005) 8:119–30. doi:10.1016/j.ccr.2005.07.004. PMID: 16098465 PMC3024718

[11] Goffart N Lombard A Lallemand F Kroonen J Nassen J Di Valentin E . CXCL12 mediates glioblastoma resistance to radiotherapy in the subventricular zone. Neuro Oncol. (2017) 19:66–77. doi:10.1093/neuonc/now136. PMID: 27370398 PMC5193023

[12] Lim DA Cha S Mayo MC Chen MH Keles E VandenBerg S . Relationship of glioblastoma multiforme to neural stem cell regions predicts invasive and multifocal tumor phenotype. Neuro Oncol. (2007) 9:424–9. doi:10.1215/15228517-2007-023. PMID: 17622647 PMC1994099

[13] Adeberg S Bostel T König L Welzel T Debus J Combs SE . A comparison of long-term survivors and short-term survivors with glioblastoma, subventricular zone involvement: A predictive factor for survival? Radiat Oncol. (2014) 9:95. doi:10.1186/1748-717x-9-95. PMID: 24758192 PMC4011838

[14] Jafri NF Clarke JL Weinberg V Barani IJ Cha S . Relationship of glioblastoma multiforme to the subventricular zone is associated with survival. Neuro Oncol. (2013) 15:91–6. doi:10.1093/neuonc/nos268. PMID: 23095230 PMC3534420

[15] Gupta T Nair V Paul SN Kannan S Moiyadi A Epari S . Can irradiation of the subventricular zone influence survival in glioblastoma? J Neuro-Oncol. (2012) 109:195–203. doi:10.1007/s11060-012-0887-3. PMID: 22555992

[16] Chen L Guerrero-Cazares H Ye X Ford E McNutt T Kleinberg L . Increased subventricular zone radiation dose correlates with survival in glioblastoma patients after gross total resection. Int J Radiat Oncol Biol Phys. (2013) 86:616–22. doi:10.1016/j.ijrobp.2013.02.014. PMID: 23540348 PMC3996451

[17] Evers P Lee PP DeMarco J Agazaryan N Sayre JW Selch M . Irradiation of the potential cancer stem cell niches in the adult brain improves progression-free survival of patients with Malignant glioma. BMC Cancer. (2010) 10:384. doi:10.1186/1471-2407-10-384. PMID: 20663133 PMC2918578

[18] Lee P Eppinga W Lagerwaard F Cloughesy T Slotman B Nghiemphu P . Evaluation of high ipsilateral subventricular zone radiation dose in glioblastoma: A pooled analysis. Radiother Oncol. (2013) 108:237–41. doi:10.1016/j.ijrobp.2013.01.009. PMID: 23462418

[19] Acharya S Wu S Ashford JM Tinkle CL Lucas JT Qaddoumi I . Association between hippocampal dose and memory in survivors of childhood or adolescent low-grade glioma. Int J Radiat Oncol Biol Phys. (2019) 105:297–305. doi:10.1093/neuonc/noz068. PMID: 30977510 PMC7594551

[20] Monje ML Mizumatsu S Fike JR Palmer TD . Irradiation induces neural precursor-cell dysfunction. Nat Med. (2002) 8:955–62. doi:10.1038/nm749. PMID: 12161748

[21] Valiyaveettil D Malik MU Joseph B Gupta T . Subventricular zone irradiation in glioblastoma: A systematic review. J Neuro-Oncol. (2020) 149:213–23.

[22] Weller M van den Bent M Tonn JC Stupp R Preusser M Cohen-Jonathan-Moyal E . European Association for Neuro-Oncology (EANO) guidelines on the diagnosis and treatment of adult astrocytic and oligodendroglial gliomas. Lancet Oncol. (2017) 18:e315–29. doi:10.1016/s1470-2045(17)30194-8. PMID: 28483413

[23] Lee J Kotliarova S Kotliarov Y Li A Su Q Donin NM . Tumor stem cells derived from glioblastomas cultured in bFGF and EGF more closely mirror the phenotype and genotype of primary tumors than do serum-cultured cell lines. Cancer Cell. (2006) 9:391–403. doi:10.1016/j.ccr.2006.03.030. PMID: 16697959

[24] Gupta T Nair V Jalali R . Stem cell niche irradiation in glioblastoma: providing a ray of hope? CNS Oncol. (2014) 3:367–76. doi:10.2217/cns.14.39. PMID: 25363009 PMC6113729

[25] Chen L Quinones-Hinojosa A Ford E McNutt T Kleinberg L Lim M . Increased radiation dose to the SVZ improves survival in patients with GBM. Int J Radiat Oncol. (2012) 84:S8. doi:10.1016/j.ijrobp.2012.07.027. PMID: 38826717

[26] Elicin O Inac E Uzel EK Karacam S Uzel OE . Relationship between survival and increased radiation dose to subventricular zone in glioblastoma is controversial. J Neurooncol. (2014) 18(2):421. doi: 10.1007/s11060-014-1424-3. Erratum in: J Neurooncol. (2014) 118(2):421. 24668610

[27] Kazmi F . Impact of subventricular zone irradiation on survival outcomes in glioblastoma patients. J Neuro-Oncol. (2014).

[28] Khalifa J Tensaouti F Lusque A Plas B Lotterie JA Benouaich-Amiel A . Subventricular zones: new key targets for glioblastoma treatment. Radiat Oncol. (2017) 12(1):67. doi:10.1186/s13014-017-0791-2 28424082 PMC5397708

