# Supplementary material for: Assessment of subventricular zone irradiation in glioblastoma patients
Source: Front Oncol. 2026 May 22;16:1818058. doi: 10.3389/fonc.2026.1818058 (PMC13236565; doi:10.3389/fonc.2026.1818058)
Supplement: Supplementary file 1 [file DataSheet1.pdf]

Study (Author/year)	Sample size	Threshold dose to SVZ	MEAN SVZ VOLUME (cc)			MEAN SVZ DOSE (Gy)			PROGRESSION FREE SURVIVAL			OVERALL SURVIVAL		
			I/L	C/L	B/L	I/L	C/L	B/L	Median, (Range), months	HR Univariate (95% CI)	P value	Median, (Range), months	HR Univariate (95% CI)	P value
**Evers et. al. (2010)**	55	>43Gy	5.05			46 +/-15.5	41+/-16.1		15 Vs 7.2		0.03			
**Gupta et. al. (2012)**	40	iSVZ > 58Gy	5.6+/-2.5	6.4+/-3		58.7Gy	53.6Gy	56.2 Gy	11, (8.9-13), 10 Vs 11		0.92	17, (11.6-22.4), 17 Vs 15		0.95
**Lee et. al. (2013)**	173	iSVZ > 59.4 Gy	4.3+/-1.3	5+/-1.6		49.2 Gy	35.2 Gy	60.1 Gy	10.4, (0.1-71.3)	0.56 (0.32-0.98)	0.042	19.6 (4.4-104)	0.67 (0.38-1.19)	0.173
**Chen et. al. (2013)**	116	iSVZ >/= 40Gy	7.05 (2.99-4.2)	7.91 (4.18-14.6)	14.76 (5.37-28.3)	48.7 (1.96-60)	34.4 (1.59-60)		41.5 (1.77-60)	0.824 (0.506-1.34)	0.434		0.926 (0.570-1.5)	0.754
**Elicin et. al. (2014)**	60	cSVZ >59.2Gy	5.2+/-2.4	6.4+/-2.3	11.6+/-4.2	58.8+/-6.5	44.9+/-15.9	51.9+/-10.4	9.5 (7.7-11.1), 10.37 Vs 7.1	2.42 (1.18-4.71)	0.018	19.27 (12.77-25.23)	4.83 (1.71-13.97)	0.004
**Adeberg et. al. (2014)**	65	iSVZ >40	14.05(8.41-22.8)	14.5 (8.68-23.8)		58.7Gy	53.6Gy	6.2Gy	11, (8.9-13), 10 Vs 11		0.02	17, (11.6-22.4), 17 Vs 15		0.95
**Kusumawidjaja et. al. (2014)**	72	iSVZ- 70 (DE) Vs 60				60.6 (33.4-69.8)	39.5 (19.4-61.2)	49.1 (28.3-64.3)	7.1, (5.6-9.6), 7.1 Vs 11.1	0.95 (0.9-1)	0.052	15.2 (11-18.6) Vs 18.4	1.03 (0.97-1.1)	
**Khalifa et. al. (2016)**	43	bSVZ > 40Gy	5 (3.4-11)	5.5 (3.4-9.6)	10. (6.8-20.6)	51.3 (17.9-61.4)	15.4 (1.4-48.7)	35 (10.8-51.8)	6.5 (4.4-9.3)			22.7 (14.5-26.2)		
**Our study (2024)**	**28**	**iSVZ > 52.14**	**7.9 (6.02-10.39)**	**7.89 (5.88-10.03)**	**15.57 (11.61-22.79)**	**50.7 Gy**	**38.8 Gy**	**44.98**	**6.5, (1.44-14), Vs 6**	**0.08**	**0.78**	**13 (4.6-18.35), 13**	**4.04**	**0.04**
